# Cerebrospinal fluid chemokine patterns in children with enterovirus 71-related encephalitis

**DOI:** 10.1038/s41598-018-19988-6

**Published:** 2018-01-26

**Authors:** Jinling Liu, Shuxian Li, Chunyan Cai, Yingchun Xu, Yuan Jiang, Zhimin Chen

**Affiliations:** 1 Department of Pulmonology, The Children’s Hospital, Zhejiang University School of Medicine, Hangzhou, Zhejiang, 310003 China; 2 Department of infectious disease, Hangzhou Children’s Hospital, Hangzhou, Zhejiang, 310014 China

## Abstract

Enterovirus 71 (EV71) is a major pathogen that causes hand, foot and mouth disease (HFMD) as well as neurological complications, such as encephalitis. The chemokines involved in the migration of leukocytes have increasingly been implicated in infectious diseases of the central nervous system. Few studies have evaluated the levels of chemokines in HMFD children with EV71-related encephalitis. In the present study, we evaluated the cerebrospinal fluid (CSF) levels of the chemokines IL-8, RANTES, MIG, MCP-1 and IP-10 in 99 children with EV71-related encephalitis and 22 children with febrile convulsion (FC). We found that the concentrations of IL-8, RANTES, MIG and IP-10 were significantly higher in HFMD children with encephalitis compared to patients with FC. Additionally, these four chemokines were dramatically reduced during convalescence. Inversely, the level of MCP-1 was lower in encephalitis patients than FC patients and was not significantly reduced during convalescence. Additionally, MIG was strongly correlated with IP-10 in encephalitis patients. Furthermore, the area under the ROC curve (AUC) of CSF MIG and IP-10 in distinguishing encephalitis from FC were 0.869 and 0.876, and the corresponding sensitivities/specificities were 67.7%/100.0% and 67.7%/95.5%, respectively. In conclusion, our results indicate that chemokines play important roles in the pathogenesis of EV71 infection.

## Introduction

Enterovirus 71 (EV71) is one of the major agents that causes hand, foot and mouth disease (HFMD) in infants and young children, which is characterized by fever, ulcers in the oral mucosa and vesicles on the hands and feet^1^. Although EV71 infection in most cases is self-limited, it can also induce severe neurological complications, such as aseptic meningitis (AM), encephalitis, brainstem encephalitis (BE), pulmonary edema (PE) and other rarer manifestations^1^. These neurological complications can sometimes lead to permanent neurologic sequelae and even death, and fatal PE or cardiorespiratory failure is thought be the main disease process in fatal cases^1^. It has also been postulated that dysregulation of inflammatory responses and cellular immunity are possibly responsible for this pathogenesis^2–4^. For instance, an *in vitro* study conducted by Lu *et al*. showed that EV71 inhibited the cellular type I IFN response by targeting a subunit of the IFN receptor, IFNAR1^2^. An animal study revealed that both the lymphocyte and antibody responses reduced the mortality and tissue viral loads of EV71-infected mice^3^. In a clinical study, it was shown that elevated cytokine production (including IL-10, IL-13 and IFN-γ) and lymphocyte (including CD4 + T cells, CD8 + T cells, natural killer cells) depletion were responsible for EV71 infection^4^. Unfortunately, to date, there is no effective vaccines or therapy available for EV71 infection. Therefore, developing a better understanding of the neuropathogenesis of EV71 infection is of the utmost importance in developing a more effective clinical therapy for treating HMFD patients.

Chemokines are a group of small secreted proteins that have been shown to induce directed chemotaxis in response to inflammation^5^. The most investigated chemokines belong to the CC and CXC families, which are classified by the relative position of the first consensus cysteines (either separated by a non-conserved amino acid or next to each other). CC chemokines include regulated upon activation normal T cell expressed and secreted (RANTES, also termed CCL5) and monocyte chemoattractant protein-1 (MCP-1, also termed CCL2), while interferon-γ-inducible protein 10 (IP-10, also termed CXCL10), monokine induced by IFN-γ (MIG, also termed CXCL9) and interleukin-8 (IL-8, also termed CXCL8) belong to the CXC family^5^. Although they were initially characterized as being important in inflammation by targeting leukocytes, chemokines are now considered to be crucial mediators that play fundamental roles in both the physiological and pathological immune responses^5^. For example, the concentration of RANTES is higher in patients with a bacterial infection (including sepsis, community-acquired pneumonia, skin abscess) than controls with no infection, indicating that RANTES may be involved in the pathogenesis of bacteria^6^. In the same study, a unique pattern of immune response with high levels of IP-10 but low levels of MCP-1 were found in patients with sepsis^6^. IP-10, MIG and IL-8 were significantly elevated in the sputum of asthma patients compared to healthy controls^7^. Similarly, the plasma levels of IP-10, MCP-1, MIG and IL-8 were significantly higher in patients with PE than those with uncomplicated BE, indicating overexpression of the chemokine cascade, which appears to play an important role in the immune response to EV71 infection^8^. However, few studies have evaluated the levels of chemokines in the cerebrospinal fluid (CSF) of HMFD children. Additionally, encephalitis is the common neurological complication of EV71 infection; little is known about the roles of chemokines in encephalitis, and the key mediators underlying this inflammatory response remain unclear. Moreover, despite the severe consequences, early recognition of HFMD patients with neurological complications is important for selecting optimal intensive care, which might prevent disease progression.

Therefore, the purpose of this study was to (i) evaluate the CSF levels of the chemokines IL-8, RANTES, MIG, MCP-1 and IP-10 in HFMD patients with encephalitis; (ii) identify the possible interrelationships between the chemokines and CSF parameters; and (iii) investigate their potential role in HFMD as biomarkers reflecting pathogenesis, which may pave the way for new therapeutic targets.

## Results

### Patient characteristics and laboratory variables

This study included 99 HFMD patients with EV71-related encephalitis and 22 FC patients as controls. The baseline characteristics of the study population are listed in Table 1. In brief, there were no significant differences between encephalitis and FC patients regarding age, gender, and cell number in blood. However, encephalitis patients were more likely to have a longer duration of fever and hospital stay, have higher blood glucose, and have higher WBC and protein concentrations in CSF compared to children with FC.

**Table 1:** Comparison of the demographic and clinical characteristics between patients with EV71-related encephalitis and those with simple FCs.

Category, characteristic	Febrile convulsion (n = 22)	Encephalitis (n = 99)	*P*
Age (months)	26.68 ± 16.84	29.26 ± 13.20	0.433
Gender (male/female)	13/9	60/39	0.895
Hospital stays (days)	6.23 ± 2.07	9.60 ± 2.45	<0.001
Fever duration before admission (days)	1.61 ± 1.17	2.49 ± 1.55	0.007
Fever duration during hospitalization (days)	1.76 ± 1.19	2.44 ± 1.28	0.003
Total fever duration (days)	3.37 ± 1.66	4.92 ± 1.61	<0.001
Blood routine analysis			
WBC (×10^9^/L)	13.10 ± 3.86	11.53 ± 3.82	0.084
Neutrophil (%)	56.69 ± 22.12	59.47 ± 16.01	0.793
Lymphocyte (%)	31.65 ± 18.42	33.38 ± 14.50	0.441
Hemoglobin (g/L)	119.77 ± 10.76	121.09 ± 9.07	0.553
Platelet count (×10^9^/L)	299.23 ± 65.56	309.33 ± 73.81	0.555
Blood glucose (mmol/L)	5.27 ± 1.47	6.05 ± 1.62	0.043
CSF variables			
WBC (×10^6^/L)	4.14 ± 2.66	125.58 ± 123.68	<0.001
Neutrophil (%)	—	49.54 ± 24.96	—
Lymphocyte (%)	—	45.03 ± 24.65	—
Total protein (g/L)	166.86 ± 78.11	267.34 ± 123.95	<0.001
Glucose (mmol/L)	3.85 ± 0.75	5.11 ± 11.44	0.592
Chloride (mmol/L)	124.5 ± 4.46	122.07 ± 13.21	0.894

### CSF chemokine profiles in the acute and recovery phases of EV71-related encephalitis

**Figure 1:**
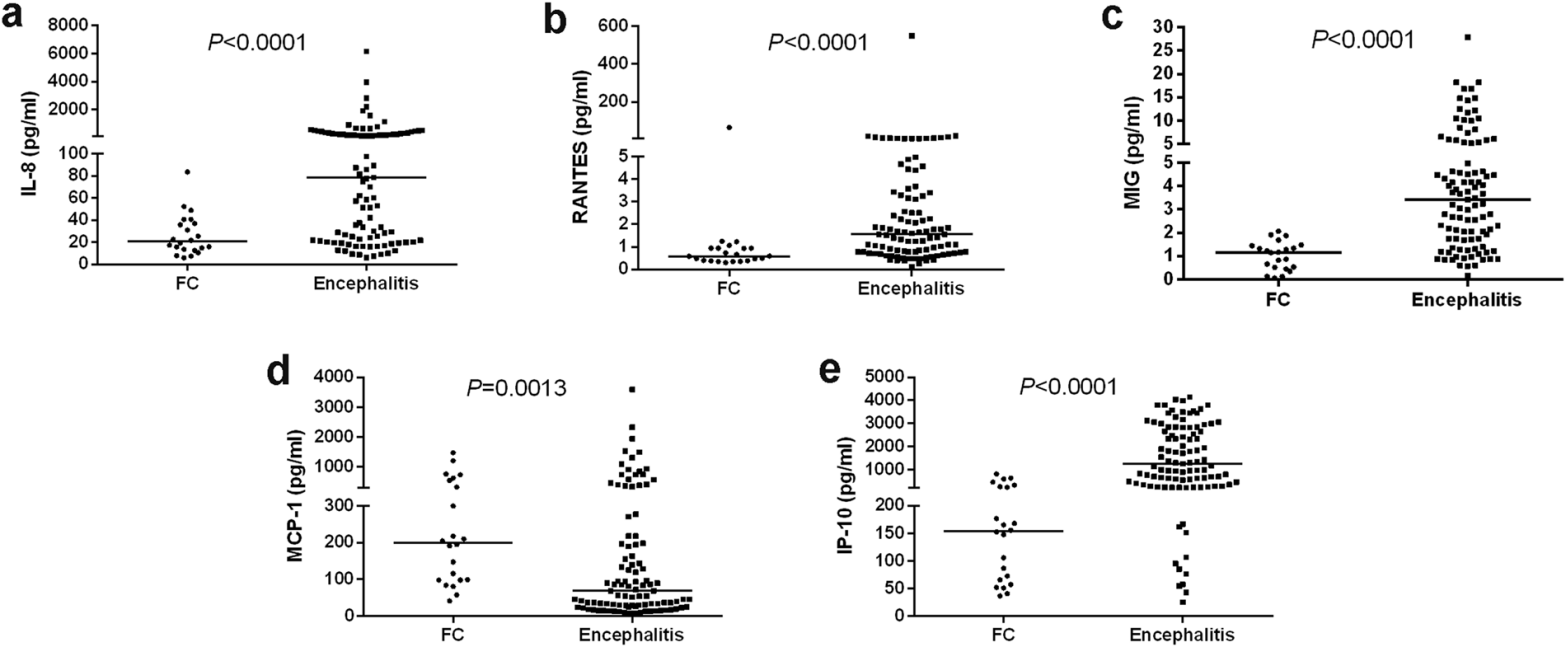
CSF chemokine concentrations in EV71-related encephalitis patients and febrile convulsion (FC) patients in the acute stage. (**a**) IL-8; (**b**) RANTES; (**c**) MIG; (**d**) MCP-1; (**e**) IP-10.

The CSF chemokine levels of IL-8 (78.40 (21.73–267.34) pg/ml), RANTES (1.56 (0.77–3.30) pg/ml), MIG (3.42 (1.72–5.34) pg/ml) and IP-10 (1238.82 (391.55–2812.31) pg/ml) were significantly higher in patients with encephalitis in the acute stage compared to those with FC (20.56 (13.28–37.98) pg/ml, 0.59 (0.38–0.94) pg/ml, 1.16 (0.50–1.46) pg/ml, 154.12 (63.48–264.60) pg/ml, respectively) (Fig. 1a,b,c,e). The CSF MCP-1 levels, on the contrary, were significantly lower in the encephalitis group (69.06 (29.37–217.59) pg/ml) compared to the FC group (199.84 (98.13–571.13) pg/ml) (Fig. 1d).

Furthermore, we investigated the association between the chemokine levels in the acute phase and fever duration before admission. There was a positive correlation between RANTES and fever duration before admission (r = 0.469, *p* < 0.001). However, IL-8 (r = −0.398, *p* < 0.001) and MCP-1 (r = −0.294, *p* = 0.003) were negatively correlated with fever duration before admission. In addition, neither MIG (r = 0.114, *p* = 0.260) nor IP-10 (r = −0.139, *p* = 0.170) were associated with fever duration before admission.

**Figure 2:**
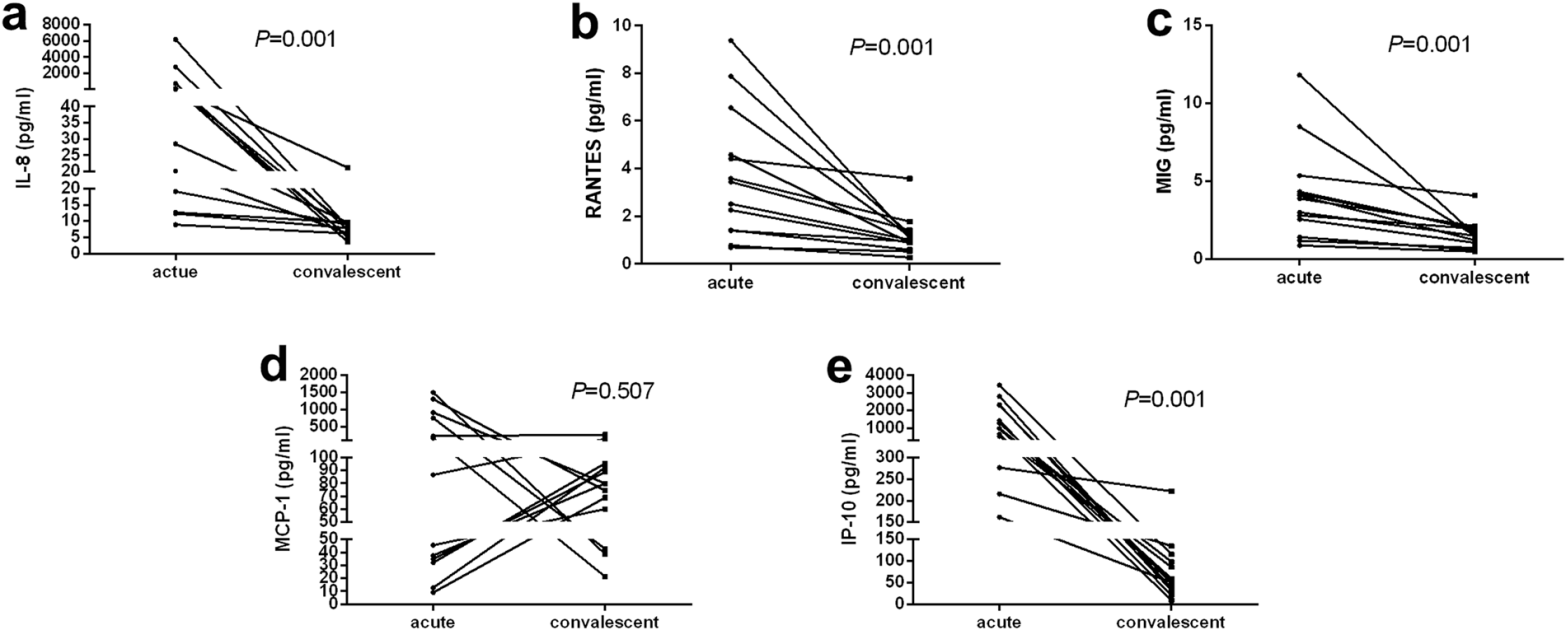
CSF chemokine concentrations in EV71-related encephalitis patients in the acute and convalescent stage. (**a**) IL-8; (**b**) RANTES; (**c**) MIG; (**d**) MCP-1; (**e**) IP-10.

Compared with the acute stage, the CSF chemokine levels of IL-8 (77.54 (15.90–473.87) pg/ml vs. 7.89 (5.73–9.28) pg/ml), RANTES (3.44 (1.40–5.57) pg/ml vs. 1.00 (0.74–1.33) pg/ml), MIG (3.87 (1.97–4.83) pg/ml vs. 1.48 (0.86–1.95) pg/ml) and IP-10 (980.79 (406.20–2314.40) pg/ml vs. 56.07 (34.51–106.77) pg/ml) significantly decreased in the convalescent stage (Fig. 2a,b,c,e) of encephalitis in 13 patients, except for MCP-1 (86.60 (33.42–831.52) pg/ml vs. 79.86 (51.36–93.89) pg/ml) (Fig. 2d).

### The association between the CSF chemokine levels and CSF cytology parameters in encephalitis patients

**Figure 3:**
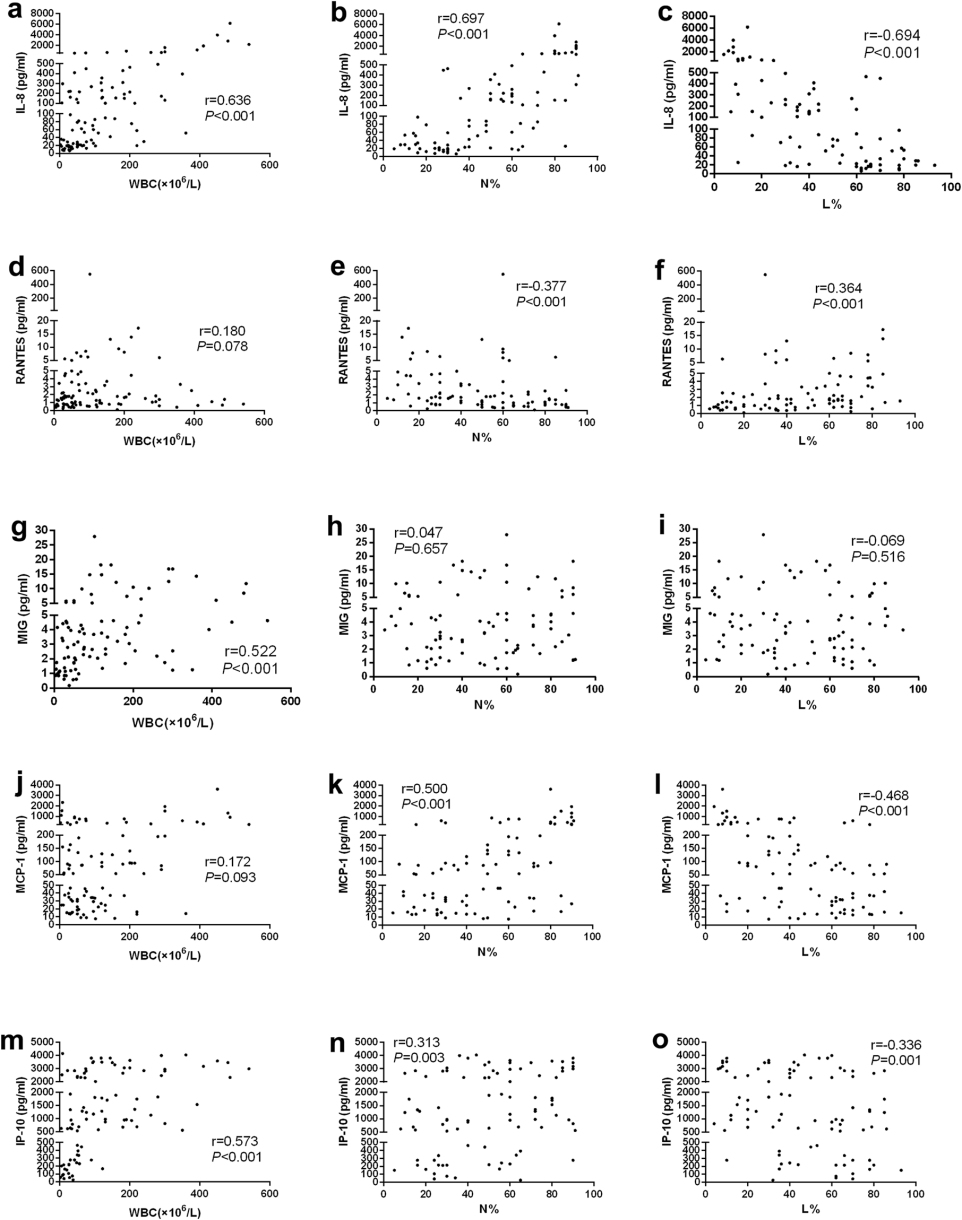
Correlation analysis of CSF chemokine concentrations with CSF cytology variables in encephalitis patients depicted as scatter plots.

In encephalitis patients, all of the CSF cytology variables (including WBC, neutrophil percentages and lymphocyte percentages) were strongly associated with the CSF IL-8 levels (Fig. 3a–c). Although the CSF MIG and IP-10 concentrations correlated well with the CSF WBC (Fig. 3g,m), they showed poor or no correlation with the neutrophil and lymphocyte percentages (Fig. 3h,i,n,o). By contrast, a statistically significant correlation between the CSF RANTES, MCP-1 levels and neutrophil and lymphocyte percentages (Fig. 3e,f,k,l) were detected, but no correlation with the CSF WBC was found (Fig. 3d,j). We did not find any correlation between the CSF chemokine levels and cytology variables in FC patients (Supplementary Table S1).

### Clinical diagnostic values of CSF chemokines for HFMD patients with EV71-related encephalitis

**Figure 4:**
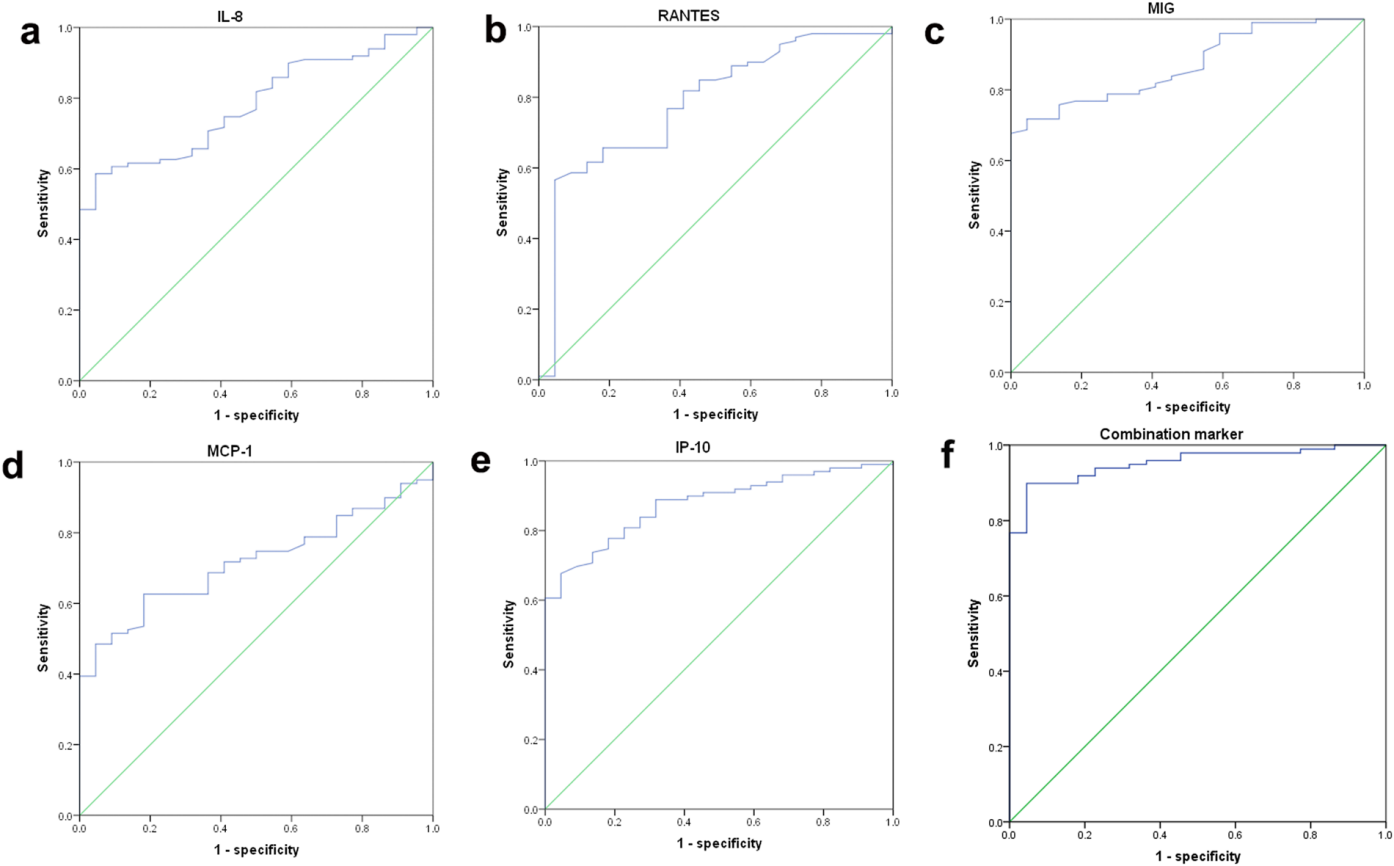
ROC analysis of CSF chemokines in the encephalitis and FC groups. (**a**) IL-8; (**b**) RANTES; (**c**) MIG; (**d**) MCP-1; (**e**) IP-10; (**f**) combination marker of five chemokines.

Given the marked variation of CSF chemokines in HFMD patients with encephalitis, the performance characteristics of CSF chemokines in differentiating encephalitis from FC were evaluated by ROCs. As shown in Table 2 and Fig. 4, ROC analyses showed that IP-10 and MIG yielded a significantly increased area under a ROC of 0.876 and 0.869, respectively, compared with IL-8 (0.781), RANTES (0.783), MCP-1 (0.716). The Youden-index-calculation provide a sensitivity of 67.7% and specificity of 95.5% for a cut-off of 621.79 pg/ml for IP-10 (Fig. 4e). When the cut-off point of MIG was 2.10 pg/ml, its sensitivity and specificity were 67.7% and 100%, respectively (Fig. 4c). Similarly, IL-8, RANTES and MCP-1 had 58.6%, 56.6% and 62.6% sensitivity, and 95.5%, 95.5% and 81.8% specificity, when the optimal cut-off values were 52.71 pg/ml, 1.26 pg/ml and 97.24 pg, respectively (Fig. a,b,d). Subsequently, a combination marker of these five chemokines was evaluated by ROC analysis, which had an area under the ROC curve of 0.953 (Table 2). Its sensitivity and specificity were 89.9% and 95.5%, respectively (Fig. 4f). Hence the diagnostic efficiency of the combination marker was superior to that of the single chemokine.

**Table 2:** ROC analysis of CSF chemokines in HFMD patients with encephalitis.

Variable	Area	Standard error	P value	95% CI
IL-8/CXCL8	0.781	0.044	<0.001	0.694–0.867
RANTES/CCL5	0.783	0.054	<0.001	0.678–0.888
MIG/CXCL9	0.869	0.033	<0.001	0.804–0.934
MCP-1/CCL2	0.716	0.047	0.002	0.624–0.809
IP-10/CXCL10	0.876	0.033	<0.001	0.812–0.940
Combination marker	0.953	0.018	<0.001	0.917–0.989

### The internal association between chemokines

**Figure 5:**
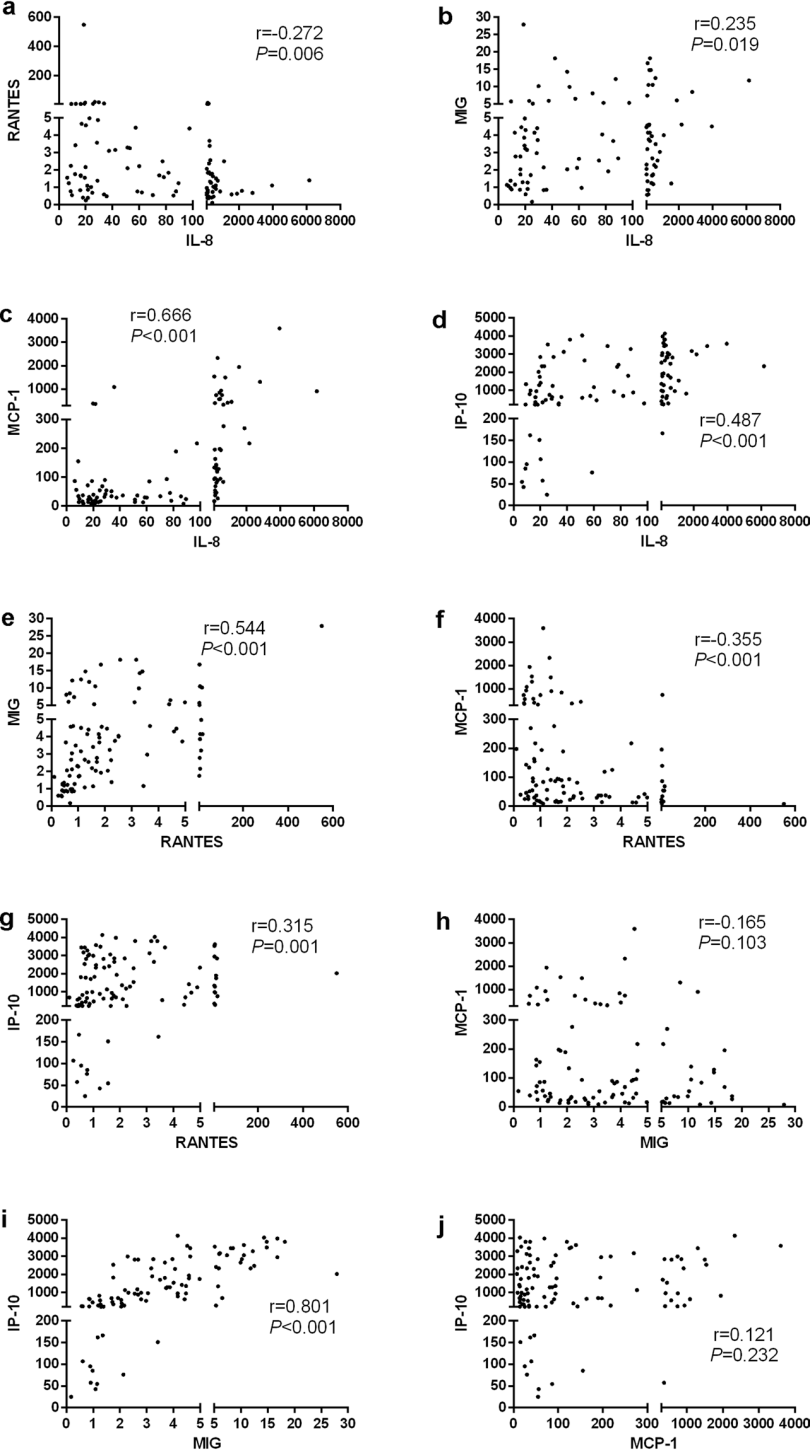
Interrelation analysis between the chemokines in encephalitis patients depicted as scatter plots.

To corroborate these observations, correlation and interrelation analysis was performed within these CSF chemokines. As shown in Fig. 5, IL-8 was positively correlated with MIG, MCP-1 and IP-10, whereas it was negatively correlated with RANTES. IL-8 was strongly associated with MCP-1 (Fig. 5c). Likewise, MIG, MCP-1 and IP-10 were associated with RANTES (Fig. 5e–g). Of note, there was a strong correlation between MIG and IP-10 (Fig. 5i). Furthermore, we did not find any correlation between MIG and MCP-1 (Fig. 5h). Similarly, there was no significant correlation between MCP-1 and IP-10 (Fig. 5j).

## Discussion

Increasing evidence suggests that cellular and humoral immune dysfunction is involved in the exacerbation of HFMD^8–10^. Still, the effect of EV71 infection on the host’s adaptive immunity, especially chemokines, is not well understood. In the present study, the chemokines IL-8, RANTES, MIG, MCP-1 and IP-10 were found to be significantly changed in the CSF of HFMD patients in the acute stage of EV71-related encephalitis (Fig. 1). Our finding suggests that chemokines, which function in the selective recruitment of leukocyte populations, are involved in the pathology of the central nervous system (CNS) EV71 infection. The increased expression levels of IL-8, IP-10 and MIG are consistent with those of previous studies^8,11^. In addition, we found chemokines that were correlated with each other (Fig. 5), in accordance with the result of string bioinformatics analysis^12^, which showed that these chemokines act in cooperation with each other rather than alone (Supplementary Fig. S1). Likewise, a cytokine storm has been considered to be the main trigger of EV71-related cardiopulmonary collapse^13^. Taken together, this evidence implied that the dysregulation of the chemokine cascade in the CNS appears to be important regarding the elicitation of the inflammatory response to EV71 infection.

Chemokines are highly involved in the regulation of leukocyte migration and inflammation^5^, which may provide an explanation for the significant correlation between chemokines and CSF cytology. MIG and IP-10 are Th1 cell attractants, while MCP-1 is Th2 chemokine and RANTES can recruit both Th1 and Th2 cells^5^. Previous studies have shown that HFMD is a Th1-driven disease^4,9^. In line with this finding, we also showed that Th1 chemotactic factors (including MIG, IP-10 and RANTES), rather than MCP-1, are potent chemoattractants of Th2 and are significantly elevated in the acute phase. Thus, we assumed that increased CSF MIG, IP-10 and RANTES in children with encephalitis may result in enhancement of Th1 lymphocyte recruitment into the CNS. Accordingly, decreased expression of the Th2 chemokine MCP-1 in encephalitis patients may contribute to a diminished antagonizing effect on Th1 cytokine production and hence intensify Th1 predominance. Interestingly, we revealed that MIG, IP-10 and RANTES were decreased in the recovery phase, while no significant differences were observed for MCP-1. Together, these data imply that Th1 chemoattractants predominate after EV71 infection and contribute to host defense by promoting a protective Th1 response.

MIG and IP-10 are IFN-inducible CXC chemokines and are potent chemoattractants for activated T cells, memory T cells and natural killer cells by signaling through the CXCR3 receptor^14^. This could explain why MIG was strongly and positively correlated with IP-10, reflecting a temporal co-operation between CXCR3 ligands. An abundance of data demonstrated that MIG and IP-10 are induced in a diverse spectrum of neuroimmune diseases that influence the CNS^14^. Shen *et al*. observed that overexpression of IP-10 increased the MIG levels, reduced the viral burden in multiple tissues and increase the survival of mice after EV71 infection^15^. Consistently, IP-10 and MIG were significantly increased in the present study. Since chemokines are produced by CNS cells within hours of infection^5^, we suppose that the increased chemokine levels in patients with encephalitis may mediate early regulation and recruitment of protective immunity, reducing tissue damage. Likewise, it was reported that IP-10(−/−) mice infected with a neurotropic mouse hepatitis virus had reduced MIG levels that failed to control viral replication in the brain^16^. Kuang Y *et al*. showed that IP-10 enhanced the blood-brain barrier (BBB) and attenuation of rabies virus (RABV)^17^. This evidence suggests that early expression of IP-10 or MIG within the CNS after virus infection is important in initiating and maintaining a protective immune response. However, it has been implicated that herpes simplex virus (HSV), severe acute respiratory syndrome (SARS) and lymphocytic choriomeningitis virus (LCMV) can aggravate disease^18–20^. In detail, exaggerated activation of IP-10 and MIG have been proposed to be associated with adverse outcomes (intensive care unit admission or death) of SARS^18^. Increased secretion of IP-10 in the eye facilitates the spread of HSV to other restricted sites within the eye^20^. IP-10 regulates the severity of the LCMV-induced inflammatory process, resulting in fatal meningoencephalitis^19^. Collectively, these studies demonstrate that IP-10/MIG have subtle and perhaps divergent roles in different virus infection.

Of note, when analyzing the CSF levels of IP-10 and MIG, as well as of other potential biomarkers for encephalitis, we found that IP-10 had the highest potential for distinguishing encephalitis patients from FC patients with high sensitivity and specificity, followed by MIG. Similarly, a previous study showed that the CSF to plasma ratio for MIG tended to increase with the increasing severity of disease and that the CSF IP-10 levels in patients with EV71 BE were significantly higher than the plasma levels in control subjects^8^. Moreover, no established antiviral treatment is available for HFMD. Therefore, our results indicated that IP-10 and MIG might be novel biomarkers to diagnose encephalitis, which may guide patient treatment in clinical practice. Although, the significant difference of IP-10 and MIG had been determined in the CSF, the number of detected cases was not large enough to have power as a solo diagnostic biomarker for encephalitis. Moreover, we performed ROC analysis to analyze the diagnostic efficiency of the markers for encephalitis and showed that the sensitivities of CSF IP-10 and MIG were low (67.7% and 67.7%). However, the combination marker of the five chemokines had a significant elevation of the area under the ROC of 0.953, and its sensitivity in detecting encephalitis was 89.9%. Therefore, the combination of these five chemokines could be applied to clinical diagnosis of encephalitis, which may potentially serve to monitor disease progression, and guide clinical diagnosis and treatment.

The levels of MCP-1, however, presented a different pattern with low concentrations in patients with encephalitis compared to FC patients. Similarly, Wang *et al*. showed that the ratio of mean CSF to plasma levels for MCP-1 tended to fall with increasing disease severity^8^. These results suggest that MCP-1 may contribute to the overwhelming disease process. Inversely, it was shown that the plasma MCP levels elevated with increasing disease severity^8^. This could be explained by the difference in the immune response between the systemic and local inflammatory response. Interesting, although the MCP-1 levels decreased in convalescence, there was no statistically significant difference of the MCP-1 levels between the acute and recovery phases. This may be associated with the small paired sample size. To better elucidate this aspect, it would be interesting to determine the MCP-1 levels over a larger scale and at more than one time-point.

This study was limited by the small paired sample size and single time-point for most cases. Moreover, the control group did not include healthy children without underlying diseases because of ethical issues. Despite the limitations associated with the study group populations, the present findings enhance our understanding of EV71 pathogenesis and provide strategies for a better biomarker/therapeutic target for EV71 infection.

In conclusion, we detected that the CSF levels of four chemokines, IL-8, RANTES, MIG, and IP-10, were significantly elevated in HMFD patients with EV71-related encephalitis. Importantly, the results of the correlation and ROC curve analyses indicated that CSF IP-10 and MIG may have clinical diagnostic values in encephalitis. Additionally, further studies are needed to validate whether IP-10 and MIG could be therapeutic targets for EV71 infection. Thus, our findings provide important insights into the roles of some chemokines in the pathogenesis of EV71 infection.

## Materials and Methods

### Study subjects

This study was carried out at Children’s Hospital, Zhejiang University School of Medicine and Hangzhou Children’s Hospital between April and August 2013 and was approved by the Ethics Committees of both hospitals. All methods were conducted in accordance with the Declaration of Helsinki. Written informed consent was obtained from the children’s parents or guardians prior to enrolment.

Patients were identified and referred for enrolment by senior physicians after a careful examinations and evaluation. Diagnosis of HFMD was determined based on a popular or vesicular rash on the hands, feet, mouth or buttocks, which was usually accompanied with fever. Encephalitis was defined by the presence of an altered level of consciousness (including lethargy, drowsiness or coma, seizures or myoclonus) with CSF pleocytosis. In addition, patients with simple febrile convulsion (FC) were enrolled as controls. Diagnosis of FC was based on the criteria described previously^21^. In brief, simple FC was defined as generalized tonic-clonic seizures occurring in the first 48 hrs of a febrile illness and lasting for less than 15 min, but without any focal signs or recurring within 24 hrs, or no associated evidence of intracranial infection or a metabolic disorder^21^.

### Sampling and data collection

At enrolment, throat swabs and rectal swabs were collected from all participants. Meanwhile, a lumbar puncture was taken to obtain an acute phase CSF sample from each patient. All clinical samples from the HFMD patients mentioned above were taken at the same time and within 3 days from HFMD symptom onset. Unfortunately, recovery phase CSF samples were only obtained from 13 HFMD patients with encephalitis. All samples were stored at −80 °C until analysis. All of the clinical data of the participants were collected by reviewing electronic medical records from both hospitals, including age, gender, hematologicalparameters, CSF cytology and CSF biochemical markers.

### Real-time RT-PCR

The EV71 genome was detected in throat or rectal specimens by real-time RT-PCR as described previously^21^. In brief, total RNA was extracted from specimens using a Viral RNA Mini Kit (Qiagen, Hilden, Germany) and reversed transcribed into cDNA with the PrimeScript RT reagent kit (Da An Gene, Guangzhou, China). qPCR reactions based on TaqMan technology were performed using a 7500 real-time RT-PCR system (Applied Biosystems, Foster, CA, USA).

### Cytometric bead array analysis of CSF chemokines

A cytometric bead array (CBA), was used to measure a panel of chemokines, namely IL-8, RANTES, MIG, MCP-1 and IP-10. In short, using 50 μl of standard and sample dilutions, the assay was performed according to the manufacturer’s recommendations with the BD CBA Human Chemokine Kit (BD Biosciences, San Diego, CA, USA), and analyzed on a FACSCalibur flow cytometer (Becton Dickinson, CA, USA).

### Statistical analysis

Statistical analysis was performed with SPSS software, version 22.0 (SPSS, Chicago, IL, USA). Variables are represented by the mean ± SD for normally distributed data or median and interquartile range for nonparametric distributed data. Accordingly, differences between groups were estimated by Student’s *t*-test for normal distributed data or the Mann-Whitney U test for nonparametric distributed data. Differences between the acute and recovery phases were analyzed by the paired *t*-test (normal distribution) or Wilcoxon rank sum test (nonparametric distribution). The correlation coefficient between the selected variables was calculated by Spearman’s rank correlation analysis. Receiver operator characteristic (ROC) curves were constructed to evaluate the performance characteristics of specific CSF chemokines in differentiating HFMD patients with EV71-related encephalitis from FC patients. A two-tailed *P* value < 0.05 was considered statistically significant.

### Data availability

The datasets generated during and/or analyzed during the current study are available from the corresponding author on reasonable request.

## Electronic supplementary material

Supplementary information
