# Supplementary material for: Cerebrospinal fluid chemokine patterns in children with enterovirus 71-related encephalitis
Source: Sci Rep. 2018 Jan 26;8:1658. doi: 10.1038/s41598-018-19988-6 (PMC5786096; doi:10.1038/s41598-018-19988-6)
Supplement: Supplementary file 1 — Supplementary information [file 41598_2018_19988_MOESM1_ESM.pdf]

Supplementary information

Cerebrospinal fluid chemokine patterns in children with enterovirus 71-related encephalitis

Jinling Liu, Shuxian Li, Chunyan Cai, Yingchun Xu, Yuan Jiang, Zhimin Chen

Table S1. Correlation between CSF chemokine concentrations (pg/ml) and CSF WBC in FC patients

Chemokine	WBC	
	r	*P*
IL-8/CXCL8	-0.03	0.896
RANTES/CCL5	0.315	0.154
MIG/CXCL9	0.253	0.256
MCP-1/CCL2	0.227	0.309
IP-10/CXCL10	0.372	0.088

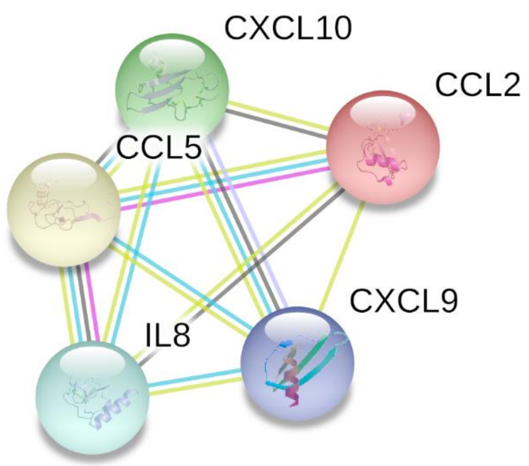

Figure S1. Protein interaction network analysis of the chemokines.
